# Locally invasive recurrence or metastasis of pheochromocytoma into the liver?—clinicopathological challenges

**DOI:** 10.1186/s12957-022-02817-6

**Published:** 2022-11-11

**Authors:** Sarah S. Tang, James W. K. Lee, Sujith Wijerethne, Shridhar Ganpathi Iyer, Susan Hue, Nga Min En, Rajeev Parameswaran

**Affiliations:** 1 Department of Surgery, Yong Loo Lin School of Medicine, National University of Singapore, Singapore, Singapore; 2 Division of Endocrine Surgery, University Surgical Cluster, National University Health System, National University Hospital, Singapore, Singapore; 3 Division of General Surgery (Hepatobiliary & Pancreatic Surgery), Department of Surgery, National University Hospital, Singapore, Singapore; 4 Department of Pathology, National University Hospital, Singapore, Singapore; 5 Alexandra Hospital, 378 Alexandra Road, Singapore, Singapore

**Keywords:** Pheochromocytoma, Adrenalectomy, Radiotherapy, Chemotherapy

## Abstract

Pheochromocytomas (PCC) are rare and functional neuroendocrine tumors developing from adrenal chromaffin cells. Predicting malignant behavior especially in the absence of metastasis can be quite challenging even in the era of improved understanding of the molecular mechanisms involved in PCCs. Currently, two histopathological grading systems Pheochromocytoma of the Adrenal Gland Scaled Score (PASS) and Grading of Adrenal Pheochromocytoma and Paraganglioma (GAPP) score are used in clinical practice, but these are subject to significant interobserver variability. Some of the most useful clinical factors associated with malignancy are large size ([4–5 cm), and genetic features such as presence of SDHB germline mutations. Local invasion is uncommon in PCC and metastasis seen in 10 to 17% but higher in germline mutations and when this occurs management can be challenging. Here, we report on a case with challenges faced by the pathologist and clinicians alike in diagnosis and management of PCC recurrence.

## Introduction

Pheochromocytomas (PCCs) are neuroendocrine tumors that arise from the chromaffin cells of the adrenal medulla. They are rare neoplasms belonging to a group of conditions known as paragangliomas with an estimated annual incidence of 0.8 per 100,000 person-years [1] and a recurrence rate of 6.5–16.5% [2]. While majority of PCCs are secretory in nature secreting excess catecholamines, a significant portion of patients are asymptomatic at diagnosis due to increased accessibility to imaging [3, 4], and genetic testing [5].

PCCs are extremely rare tumors, occurring in fewer than 0.2% of patients with hypertension [6, 7] and have an incidence of 0.8 per 100,000 person-years in the general population which peaks during the fourth and fifth decades of life [8]. Approximately 60% of these tumors are sporadic, with the rest due to germline mutations in susceptibility genes as seen in disorders such as von Hippel-Lindau (VHL) syndrome, multiple endocrine neoplasia type 2 (MEN2), and neurofibromatosis type 1 (NF1) or due to various somatic driver mutations [9–12]. It is generally believed that most PCCs in clinical practice are benign, with low metastatic potential [13].

The diagnostic evaluation of PCCs involves both biochemical evaluation and imaging studies. Currently, complete resection of the tumor is the only cure, and making precise tumor location is of paramount importance for the planning the surgical approach. Surgical extirpation is using laparoscopic and open approaches, based on the size and behavior of tumor, and local surgical expertise that may be available. *One of the continuing challenges is the differentiation between malignant and benign tumors on pathological examination as there are no definite differentiating features.* Even more challenging is the scenario of a local infiltrative recurrence, with there being no consensus on how best to evaluate and treat them.

Here, we discuss the pathology and treatment challenges of recurrent and locally invasive pheochromocytoma, using an illustrative clinical case.

## The case

**Fig. 1:**
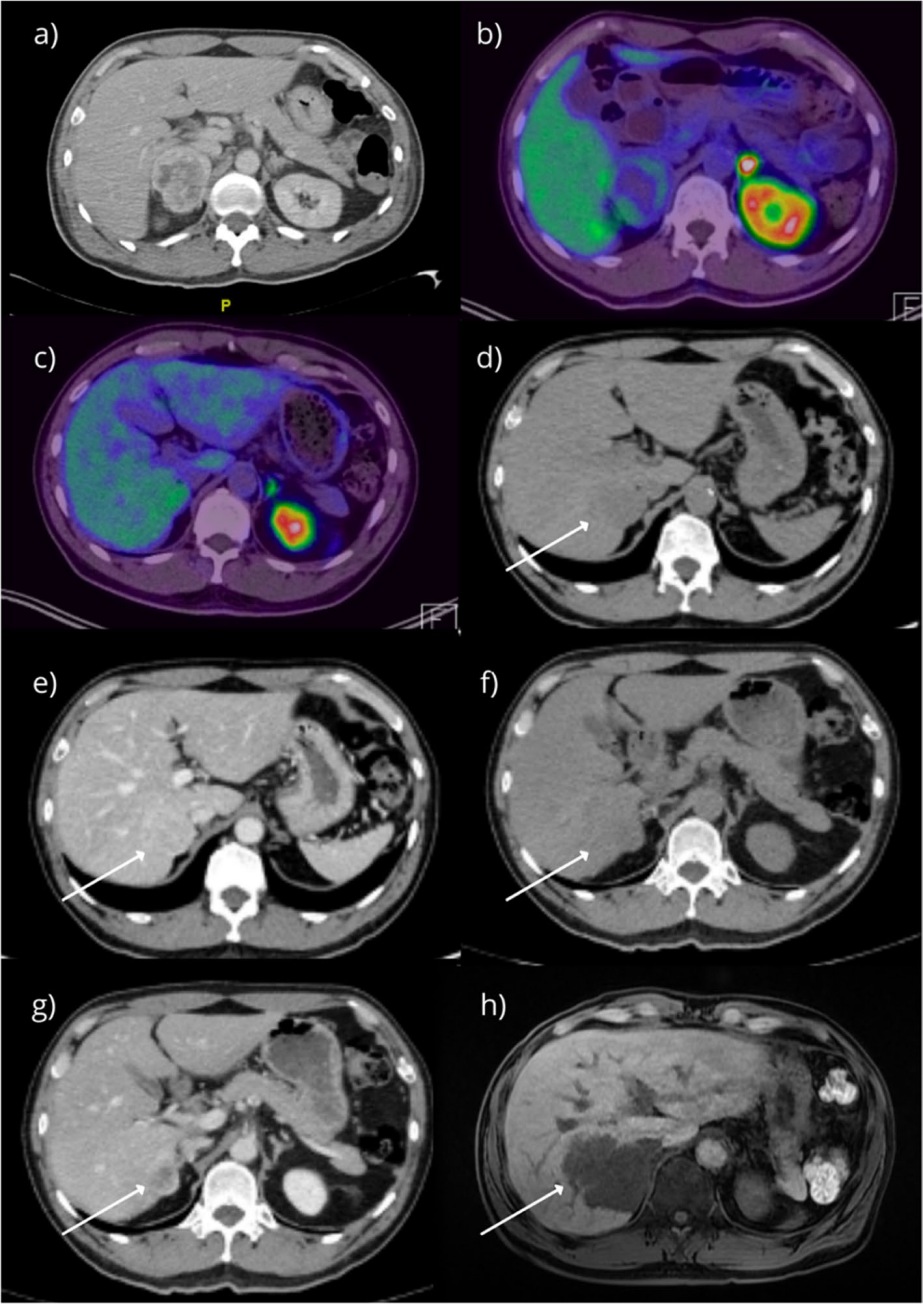
**a** CT Abdomen and pelvis, axial cut showing right adrenal lesion measuring 4.8 cm × 5.4 cm at index admission. **b**^68^Gallium-DOTANOC PET/CT showing DOTANOC avid right adrenal mass measuring 6.2 cm × 5.0 cm at index admission. **c**^68^Gallium-DOTANOC PET/CT showing DOTANOC avid right adrenal mass measuring 2.3 cm × 1.7 cm 2 years post-operatively. **d** Non-enhanced phase of adrenal CT showing mass measuring 3.2 cm × 2.9 cm (arrow) 2 years 6 months post-operatively. **e** Port-venous phase of adrenal CT showing mass measuring 3.2 cm × 2.9 cm with 77% absolute contrast washout 2 years 6 months post-operatively. **f** Non-enhanced phase of adrenal CT showing mass measuring 4.8 cm × 4.7 cm (arrow) 3 years after initial resection. **g** Porto-venous phase of adrenal CT showing mass (arrow) 3 years after initial resection. **h** MRI liver 3 years 6 months after initial resection showing mass measuring 5.2 cm × 6.7 cm × 6.2 cm in size with invasion into the right hepatic lobe involving segments 5–8 (arrow), areas of necrosis noted

A 61-year-old Chinese male patient with no significant past medical history was admitted to a tertiary referral institution following road traffic accident and underwent a pan computerized tomography (CT scan) as per trauma protocol. Abdominal imaging showed an incidental heterogeneous enhancing right adrenal mass measuring 4.8 cm × 5.4 cm with areas of internal hypodensities suggestive of necrosis (Fig. 1a). The patient was asymptomatic, with no history of hypertension, headaches, flushing, palpitations, or neurocutaneous stigmata. He had no phenotype of Cushing’s disease and there was no family history of any inherited endocrinopathy.

Biochemical investigations included pheochromocytoma screen, plasma renin/aldosterone panel and low-dose dexamethasone suppression test (LDDST) were performed for the patient, with the results as shown in Table 1. The cortisol levels were possibly secondary to stress from road traffic injuries. Functional imaging was performed using a ^68^Gallium-DOTANOC PET/CT scan which showed mild DOTANOC-avidity within a heterogeneous right adrenal mass measuring 6.2 cm × 5.0 cm with no distant uptake (Fig. 1b). Prior to surgery, the patient was started on alpha and beta blockade with Phenoxybenzamine 10 mg OM and Atenolol 25 mg OM as per institution protocol.

**Table 1:** Biochemical results at index presentation

Biochemical test	Levels (nmol/24 h)	Reference range (nmol/24 h)
Urinary adrenaline	73	3–109 nmol/24 h
Urinary oradrenaline	2075	89–473 nmol/24 h
Urinary dopamine	2270	424–2612 nmol/24 h
Urinary metanephrines	1253	325–1530 nmol/24 h
Urinary normetanephrines	20205	885–2880 nmol/24 h
Plasma aldosterone	146 pmol/L	< 445 pmol
Plasma renin	4.2 ng/ml/hr	< 0.6–3.0 ng/ml/hr
Aldosterone renin ratio	35	–
LDDST	61	< 50 nmol/L

**Fig. 2:**
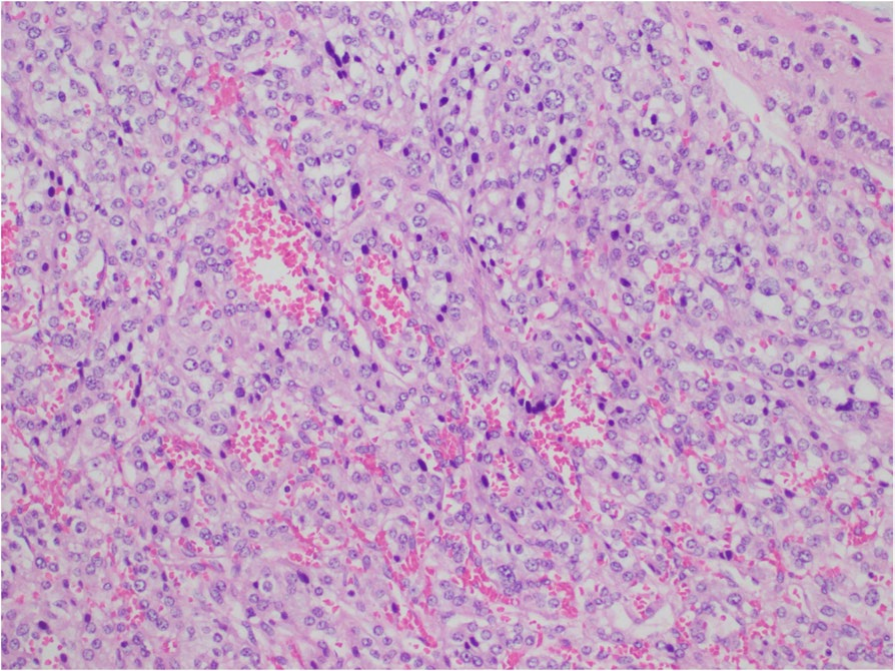
High power view shows nests of tumor cells within a richly vascular and haemorrhagic background. The tumor cells have abundant amphophilic granular to clear cytoplasm and rounded nuclei with stippled chromatin

**Fig. 3:**
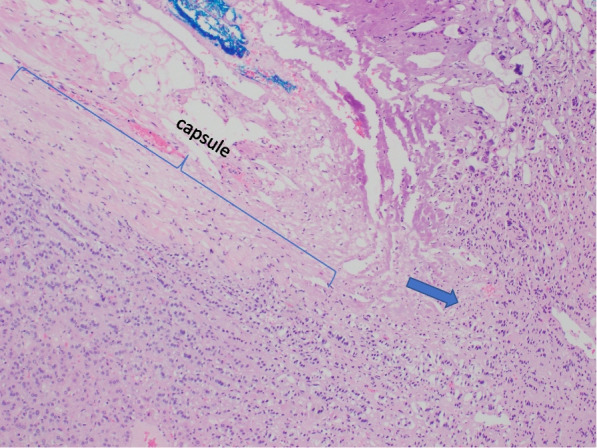
Focal capsular invasion is noted (arrowed)

A transabdominal right laparoscopic adrenalectomy was performed without any complications. Gross histological examination showed a circumscribed nodule with a variegated appearance measuring up to 6.5 cm. Microscopy showed a classical zellballen like appearance with some atypical features such as focal areas of larger nests and diffuse growth (Fig. 2), and an area of focal capsular invasion was seen with no evidence of definitive lymphovascular invasion (Fig. 3). Strong staining of the tumor cells for CD56, synaptophysin, and chromogranin was seen on immunohistochemistry, while AE1/3 and Melan-A was negative. Overall, the features were consistent with those of a pheochromocytoma, and the Pheochromocytoma of the Adrenal Gland Scoring Scale (PASS) was 3 (Table 3). A multigene genetics panel was sent for, which included screening for hereditary pheochromocytoma-paraganglioma syndrome, Von Hippel-Lindau Syndrome and Multiple Endocrine Neoplasia (MEN2), among other genes, and came back negative. A WGS was performed to evaluate the common pathological mutations associated with PCC/PPGL and none of any significance was found, including variants of unknown significance.

**Fig. 4:**
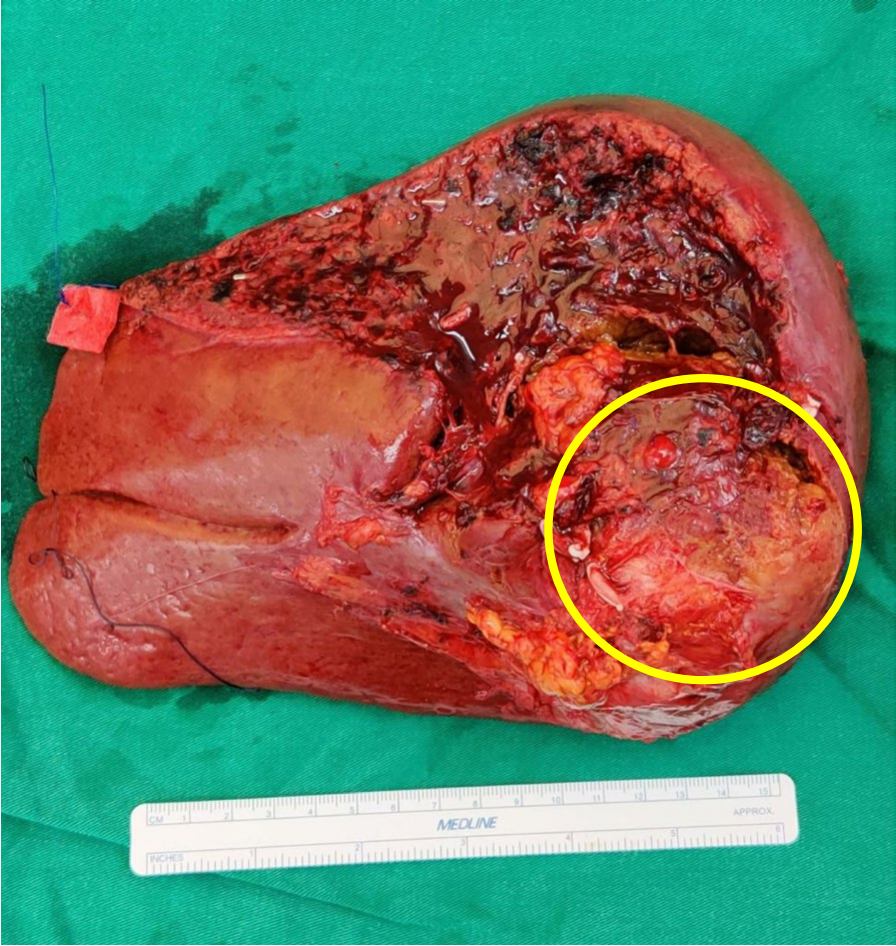
Right hepatectomy specimen, tumor measuring 5.0 cm × 7.0 cm × 7.0 cm (circled)

**Fig. 5:**
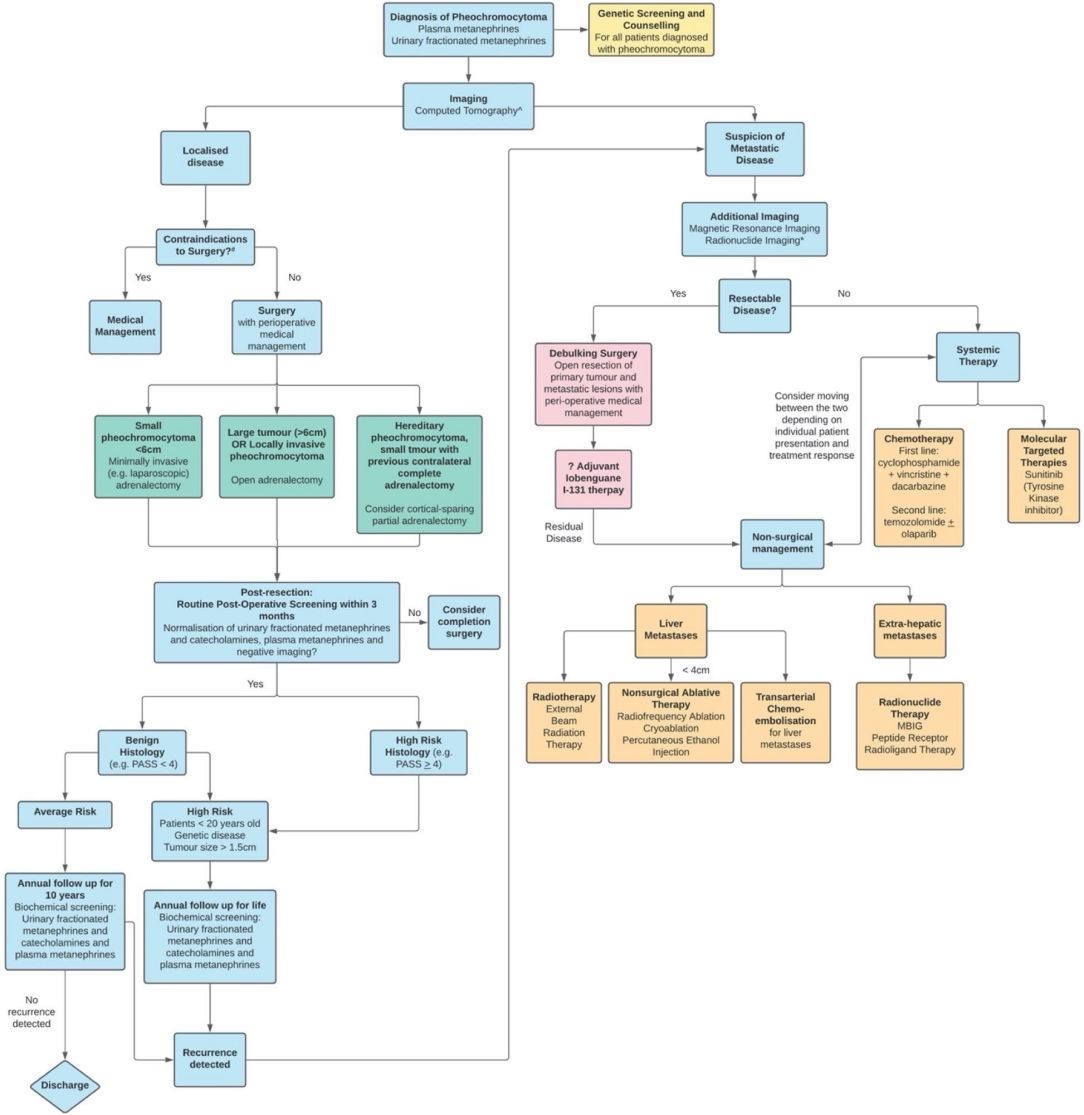
Management algorithm for pheochromocytoma and associated metastatic disease

**Fig. 6:**
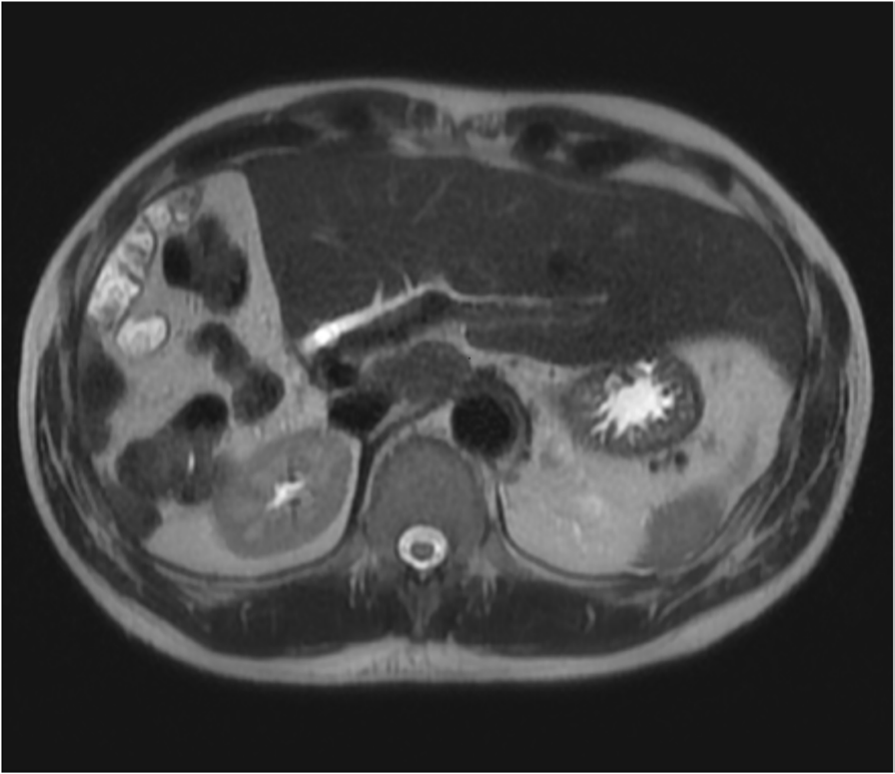
Surveillance MRI of the tumor bed showing no recurrence

The patient continued to be asymptomatic and normotensive with normal urinary metanephrines for up to 2 years following surgery. On the subsequent clinic visit the urinary biochemistry was abnormal as shown in Table 2. A repeat ^68^Gallium-DOTANOC PET/CT showed a DOTANOC-avid mass in the surgical bed superior to the surgical clips measuring 2.3 cm × 1.7 cm with a SUV max of 4.0. The tumor was inseparable from the right hepatic lobe anteriorly and abutted the intrahepatic inferior vena cava medially, with preservation of the intervening fat plane with no other DOTANOC-avid masses elsewhere (Fig. 4). The patient was restarted on Phenoxybenzamine 10 mg OM and Atenolol 25 mg OM but was PBZ was switched to Prazosin 1 mg ON following intolerance and considered for revision surgery. However, the patient opted to pursue conservative approach. Serial scans were performed and the increases in size of the tumor is shown in Fig. 5e–h. The patient underwent an open en-bloc right hepatectomy along with the tumor adherent to the inferior vena cava (Fig. 6).

**Table 2:** Biochemical results at recurrence 2 years post-index surgery

Biochemical test	Results (nmol/24 h) May 2019	Results (nmol/24 h) August 2019	Reference range (nmol/24 h)
Normetanephrine	4578	5859	885–2280
Noradrenaline	174	588	89–473

Histology of the excised tissue confirmed recurrence of the pheochromocytoma, with similar features to the initial tumor. Immunohistochemistry again showed strong expression of synaptophysin and chromogranin A. However, satellite tumors were found in the parenchyma of the liver and a focus of intravascular invasion was present. *Therefore, the clinical conundrum was that as to whether it was a recurrence in the adrenal bed invading into the liver or metastasis of the pheochromocytoma to the liver due to the presence of widespread fibrosis.* His post-operative course was uneventful, and he was discharged without any complications. The multidisciplinary tumor board recommendation was for adjuvant radiotherapy to the tumor bed, but the patient declined the treatment. Though his tumor expressed SSTR, no somatostatin analogs were considered as he was asymptomatic. He continues to be on surveillance and his last reported urinary metanephrines were normal. Post-resection, MRI scan after 1 year showed no recurrence in the tumor bed as shown in Fig. 6, and there was no evidence of distant metastasis.

## Discussion

The diagnostic criteria of ‘malignant’ pheochromocytoma remains a controversial topic. Nearly 10 to 20% of patients with PCC may develop metastasis, more commonly in patients with specific mutations [14, 15]. The 2022 WHO classification of endocrine tumors defines metastatic disease as “tumor identified at sites where normal paraganglia do not occur (i.e., histologically confirmed lymph node or bone).” [13]. Differentiating malignant tumors from benign ones is a challenging task as they may appear histologically and biochemically identical, and currently there are no markers either histological or molecular or predictive factors that can differentiate the two spectra of disease. However certain factors such as large tumor size, extra-adrenal location, increased dopamine secretion (> 3-fold increase), high Ki-67 index and *presence of SDHB mutation* (most important factor) to be associated with higher metastatic potential in PCCs [6, 16–18].

Risk-stratification scores using histological features such as the Pheochromocytoma of the Adrenal Gland Scaled Score (PASS) [19] and *Grading of Adrenal Pheochromocytoma and Paraganglioma* (GAPP) score [20] are commonly used in clinical practice to predict risk of malignancy aid decision-making. The various parameters used in the two scoring systems are shown in Table 3. Tumors with a PASS > 4 and GAPP > 3 are thought to have increased metastatic potential though with lower specificity [19, 21]. However, there remains no high-level evidence behind the use of any prognostication score. Apart from determining the malignant potential of a pheochromocytoma, the risk of recurrence is an important clinical consideration. The recurrence rate for PPGLs is estimated to be one per 100 person-years, with 40% being malignant recurrence.

**Table 3:** Comparison of PASS and GAPP scores following index and recurrent surgery

PASS feature	Points	Index surgery	Recurrent surgery
Vascular invasion	1	No	No
Capsular invasion	1	Yes	No
Invasion into periadrenal adipose tissue	1	No	Yes
Large nests or diffuse growth	2	Yes	No
Focal or confluent necrosis	2	No	No
High cellularity	2	No	No
Tumor cell spindling	2	No	No
Cellular monotony	2	No	No
Increased mitotic figures > 3/10 high power fields	2	No	No
Atypical mitotic figures	2	No	No
Profound nuclear pleomorphism	1	No	No
Hyperchromasia	1	No	No
Total score	19	3	1
GAPP feature	**Points scored**	**Index surgery**	**Recurrent surgery**
Histological pattern			
Zellballen	0	No	Yes
Large and irregular cell nest	1	No	Yes
Pseudorosette	1	No	No
Cellularity			
Low (< 150cells/HPF)	0	Yes	Yes
Moderate (150–250 cells/HPF)	1	No	No
High (> 250 cells/HPF)	2	No	No
Comedo necrosis			
Absent	0	No	No
Present	1	No	Yes
Ki67 labeling index (%)			
< 1	0	No	No
1–3	1	Yes	Yes
> 3	2	No	No
Catecholamine type			
Epinephrine type (E or E + NE)	0	No	No
Norepinephrine type (NE or NE + DA)	1	Yes	Yes
Non-functioning type	0	No	No
Total maximum score	10	3	6

*E* epinephrine, *NE* norepinephrine, *DA* dopamine

The European Society of Endocrinology defines high risk patients as young patients < 20 years old, those with a genetic disease, tumor size > 1.5 cm, or a paraganglioma who should be offered annual follow-up with biochemical screening for the rest of their lives [18]. Similarly, in a recent retrospective study involving 242 patients, features such as genetic mutation, younger age, larger tumor size, and PASS value were associated with recurrence [17]. With little ability to determine the natural history of PCC, the European Society of Endocrinology recommends follow-up with annual biochemical screening for at least 10 years in patients who have been operated on, and for lifelong annual follow-up in high risk patient groups [18, 22]. In addition, in patients with high-risk histology (such as PASS > 4 or GAPP > 3), should be considered under the high-risk screening group (Fig. 5).

The standard treatment of pheochromocytoma is complete surgical resection following medical therapy (alpha blockade–selective or non-selective). Minimally invasive adrenalectomy is recommended for most pheochromocytomas, while an open approach is preferred for large tumors > 6 cm and where there is local invasion [6] Partial cortical-sparing adrenalectomy may be considered for a small group of patients, namely those with hereditary disease who have small tumors and have previously undergone contralateral complete adrenalectomy, to prevent subsequent adrenal insufficiency [13]. In patients with metastatic disease, open resection of both primary and secondary lesions is preferred, where possible, as in the case of our patient [23].

Metastasis to the various organs is dependent on mutational status [24] and occurs via hematogenous or lymphatic routes usually to the bones, lungs, lymph nodes and liver [15]. Poor survival is associated with metastases to the liver and lungs especially in those with SDHB mutations compared to sporadic disease [24, 25]. Local therapies like radiotherapy, nonsurgical ablative therapy, and trans-arterial chemoembolization (TACE) may be considered in the treatment of liver metastasis, where surgical resection in not possible [26, 27]. External beam radiation therapy (EBRT) at doses > 40 Gy has been shown to provide symptom and local tumor control for sites other than liver such as soft tissue and bones [27]. Local ablative therapies such as radiofrequency ablation, cryoablation, and ethanol ablation are generally used in tumors < 4 cm and have been demonstrated to have up to 85% efficacy for local control and 92% for symptomatic control, making them a safe and effective treatment modality [28], whereas TACE may be useful especially for patients with multiple liver metastases. All these procedures used in local ablation may induce catecholamine surge causing hypertensive crisis, may require premedication and therefore must be closely monitored during treatment [29].

Systemic therapies also play a role in the management of unresectable disease and metastases involving organs other than the liver. ^131^I-MBIG has been shown to alleviate symptoms and stabilize tumor growth, with a study showing a complete response in 10%, partial response in 20% and a 5-year survival of 64% [30]. Sixty percent of Iobenguane I-131 avid tumors respond to MIBG, and it has been suggested that MBIG may be used in patients who have (a) unresectable progressive pheochromocytoma/paraganglioma, (b) symptoms from disease not amenable to locoregional control, or (c) a high tumor burden and few bony metastases [31].

As PCCs have been shown to express somatostatin receptor types 2 (SSTR2) and 3 (SSTR3), analogs such as DOTATOC and DOTATOC labeled with indium (^111^In), gallium (^68^Ga), yttrium (^90^Y), and lutetium (^177^Lu) have been used in both detection and therapy [32]. Studies have shown that peptide receptor radioligand therapy (PRRT) using Yttrium-90-labeled DOTA^0^-Tyr^3^-octreotide and lutetium Lu-177 dotatate achieved disease control or a partial response between 71 and 90% in patients with progressive unresectable pheochromocytoma and has a disease control rate of 71% [32, 33]. Systemic chemotherapy using a combination of cyclophosphamide, vincristine, doxorubicin, and dacarbazine is also used for patients with unresectable and rapidly progressive pheochromocytoma, especially in patients with high tumor burden or many bony metastases [34], with a higher efficacy in patients with SHB mutation [35]. A combination of cyclophosphamide, vincristine, doxorubicin, and dacarbazine is typically used [28], though some suggest that tumors with SDH mutations respond to temozolomide either as a single agent or in combination with other chemotherapeutic drugs such as streptozotozin, cisplatin, and 5-fluorouracil [29].

Recent understanding of the molecular pathways especially with kinase signaling involving cluster 2 PCCs have been shown to be associated with PCCs. Cluster 2 mutations involve germline mutations of the rearranged-during-transfection (RET) oncogene associated with MEN 2A/2B disease, neurofibromin (NF1), transmembrane protein 127 (TMEM127), Myc-associated factor (MAX) and somatic mutations of HRAS and fibroblast growth factor receptor 1 (FGFR1) genes [12]. The risk of metastasis in association with the cluster 2 mutations range between 2-12 %[12]. Targeted therapies such as Sunitinib, a tyrosine kinase inhibitor, has shown promise in the treatment of metastatic pheochromocytoma. A recent phase 2 trial in patients with progressive PPGL demonstrated a disease control rate of 83% and a median progression-free survival of 13 months [36].

## Conclusion

The diagnosis of malignancy in PCCs can be quite challenging for pathologists even in the era of improved understanding of the molecular mechanisms involved in PCCs. Equally, it can be challenging for the clinicians in deciding the best modality of treatment especially in locally invasive and metastatic disease. The need for multi-disciplinary discussion is vital in view of the multi-modal treatment options available made more difficult by a lack of clear evidence in the present literature. A clear clinical algorithm for its diagnosis, management and follow-up will aid clinicians in managing similar cases.

## Data Availability

There is no data that requires to be shared.
